# Vascular Injury to the Neck by a Bamboo Stick: A Case Report

**DOI:** 10.31729/jnma.7180

**Published:** 2022-01-31

**Authors:** Satish Vaidya, Robin Man Karmacharya, Swechha Bhatt, Bijaya Paudel, Manish Neupane

**Affiliations:** 1 Department of Surgery, Kathmandu University School of Medical Sciences, Dhuiikhei Hospital, Dhuiikhei, Kavre, Nepal; 2 Kathmandu University Schooi of Medicai Sciences, Dhuiikhei Hospitai, Dhuiikhei, Kavre, Nepai.

**Keywords:** *bamboo*, *case report*, *ligation*, *neck injuries*, *trauma*

## Abstract

Penetrating neck injuries causing rupture of sternocleidomastoid muscle along with transection of major vessels of the neck have significant morbidity and mortality due to the risk of severe hemorrhage and cerebral infarction. However, there are no universal guidelines for the management of penetrating neck injuries. Here, we report a case of a 67-year-old female with a lacerated wound on the left side of the neck with a complete transection of the left sternocleidomastoid muscle along with transection of internal jugular vein and two superficial branches of internal carotid artery following penetrating injury to the neck by a bamboo stick. It was managed by emergency wound exploration with ligation of the injured vessels with repair of sternocleidomastoid muscle. Post-operatively the hemorrhage was controlled and the patient was discharged on the fourth postoperative day. Thus, in a case of penetrating injury to the neck, prompt surgical wound exploration is beneficial.

## INTRODUCTION

Penetrating neck injury is an uncommon traumatic presentation accounting for nearly 5-10% of all traumatic cases.^1^ Common causes are brutal assaults, road traffic accidents, shotguns, and other high-velocity injuries.^1,2^ Penetrating injuries to the thigh, abdomen, and orbit are commonly reported in the literature but that to the neck by bamboo stick is rare. Clinicians must be familiar with the management protocol of this injury because they account for a mortality rate of 10%,^3^ caused mainly by cerebral infarctions and uncontrolled hemorrhage. Here we report a case of leftsided penetrating neck injury successfully managed with prompt surgical intervention.

## CASE REPORT

A 67-year-old female presented to the Emergency Department of Dhulikhel Hospital with a penetrating wound on the left side of the neck caused by a fall on a bamboo stick from a height of approximately 7 feet, two hours before the presentation. There was a history of major bleeding with blood loss of around 1.5 litre from the site of the wound. In an attempt to tampon the bleeding site, she had compressed the wound site with a towel.

**Figure 1:**
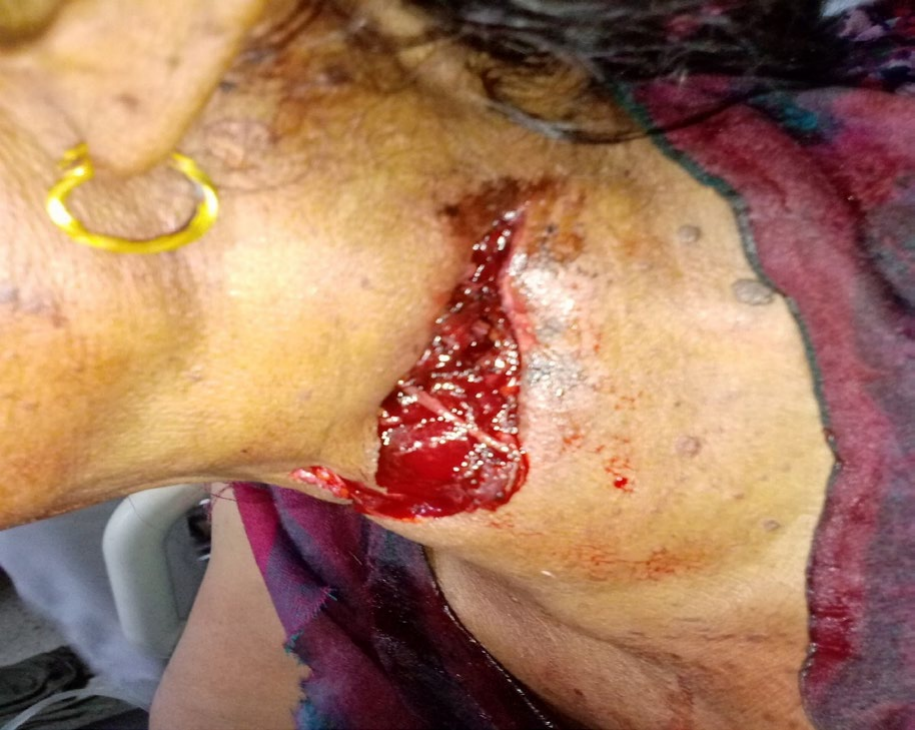
Lacerated wound on the left side of the neck measuring 7 x 3cm and depth of about 1cm with hematoma.

At the time of the presentation, she appeared ill-looking. Her blood pressure was 70/60mmHg, pulse was 63 beats per minute and oxygen saturation (SpO_2_) was 96% in room air. On examination of the wound over the left side of the neck, a lacerated wound measuring 7cm in length, 3cm in width, and 1cm depth was found extending from left mid-neck to left ear lobe laterally with the presence of hematoma (Figure 1).

**Figure 2:**
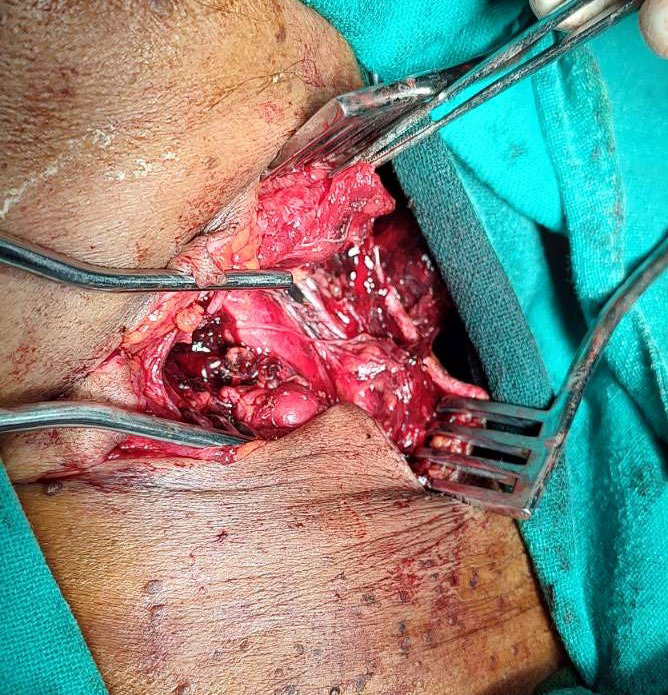
Lacerated wound showing transected vessels and muscle on the left side of the neck.

Following general examination, emergency wound exploration was done which revealed complete transection of left sternocleidomastoid muscle with transection of the left internal jugular vein and two superficial branches of the left internal carotid artery. The left vagus nerve and left phrenic nerve were intact (Figure 2).

Hematoma on the left side of the neck was evacuated with surgical repair of sternocleidomastoid muscle along with ligation of the left internal jugular vein and two superficial branches of the left internal carotid artery.

She was discharged on the 4^th^ postoperative day with analgesics and antibiotics and was advised for the regular dressing of the wound. There were no complications at the wound site and no vascular compromise.

On follow-up after a week, the patient had no signs of ischemia and infection at the site of the wound.

## DISCUSSION

Neck has complex anatomy with aerodigestive and neurovascular structures aggregated to a small region making it vulnerable when any penetrating neck injuries cross the platysma.^4^ It is thereby crucial for the clinicians to promptly manage any neck injury to avoid the risk of major hemorrhage.

For the definitive management, patients with penetrating neck injuries can be categorized as unstable with signs suggestive of injury critical injury like shock, obstruction of the airway, etc. stable with soft signs like minor bleeding, dysphagia, or asymptomatic. Similarly, according to the zone of injury involved, clinicians divide the neck into three zones: Zone I (between clavicle/ sternum and cricoid cartilage including thoracic inlet), Zone II (between zone I and zone II), and Zone III (extending from the angle of the mandible to the base of the skull), all of which have different management protocols.^4^

On presentation, a rapid assessment of the airway, breathing, and circulation is important as patients with penetrating neck injuries can decompensate rapidly.^5^ Several kinds of literature have suggested different protocols for the management of penetrating neck injuries but there is no consensus guideline for the approach.^3–7^ As exsanguination accounts for up to 50% of the mortality from penetrating neck injuries, it is indispensable for clinicians to know about bleeding control techniques along with knowledge of a surgical intervention.^4^ Initially, simple external compression is preferred, if this doesn't stop the bleeding, a Foley balloon catheter is employed for arresting the bleeding temporarily.^7^ Similar to the above approach, our patient presented to our center with compression of the wound site by a towel, which might have arrested more blood loss preventing the patient from landing into hypovolemic shock.

Aich, et al. presented a case series on penetrating injury to the neck that had no major vascular damage and was managed simply by wound toileting, followed by administration of antibiotics for prevention of secondary infection.^8^ They also suggested that injury of major vessels caused by foreign bodies should be removed only by proper wound exploration to avoid major hemorrhage following blind removal of the foreign body.^8^ Since in our case, the patient had no retained foreign body tamponing the vessels, a decision on emergency wound exploration was made.

Burgess , et al. in their study proposed that if the injury impacted a common or internal carotid artery, repair of the artery should be done before ligation despite the presence of a preoperative focal neurological deficit.^7^ However, our patient had transection of superficial branches of the internal carotid instead of the main branch, we performed the ligation of the artery over repairment aimed at arresting the major blood loss.

Despite the management of arterial injuries, bleeding might not stop if venous injuries in the neck are unnoticed.^9^ It has been indicated that isolated jugular venous injuries are usually harmless as the low-pressure venous system usually occludes without major bleeding. However, Kumar, et al. in their studies suggested the ligation of the affected vein in the advent of intraoperative finding suggestive of major bleeding resulting from injury to the jugular venous system. Same approach was implemented in our case as well. They also added that in case of sustained bilateral injury to the internal jugular vein, an attempt to repair the vein on one side should be made to minimize the risk of cerebral hypertension and edema.^9^ As the neurovascular structure of the neck has a major role in the perfusion of the brain, any neck injury should be managed rapidly with a major focus on arresting the bleeding vessels. We suggest rapid assessment with emergency wound exploration of penetrating neck injuries to avoid the risk of cerebral infarction.
